# In-hospital and day-120 survival of critically ill solid cancer patients after discharge of the intensive care units: results of a retrospective multicenter study—A Groupe de recherche respiratoire en réanimation en Onco–Hématologie (Grrr-OH) study

**DOI:** 10.1186/s13613-018-0386-6

**Published:** 2018-03-27

**Authors:** François Vincent, Marcio Soares, Djamel Mokart, Virginie Lemiale, Fabrice Bruneel, Marouane Boubaya, Frédéric Gonzalez, Yves Cohen, Elie Azoulay, Michaël Darmon, on behalf of the GrrrOH: Groupe de recherche respiratoire en réanimation en Onco-Hématologie (Group for respiratory research in intensive care in Onco-Hematology, http://www.grrroh.com/)

**Affiliations:** 1 Medical-Surgical Intensive Care Unit, Le Raincy-Montfermeil General Hospital, 10, rue du Général Leclerc, 93370 Montfermeil, France; 2 D’Or Institute for Research and Education, Rio de Janeiro, Brazil; 3 Programa de Pós-Graduação em Oncologia, Instituto Nacional de Câncer, Rio de Janeiro, Brazil; 4 Anesthesiology and Intensive Care Unit, Paoli Calmette Institute, Marseille, France; 5 Medical Intensive Care Unit, Saint-Louis University Hospital, AP-HP, Paris, France; 6 Medical-Surgical Intensive Care Unit, Mignot Hospital, Le Chesnay, France; 7 Clinical Research Unit, Avicenne University Hospital, AP-HP, Bobigny, France; 8 Medical-Surgical Intensive Care Unit, Avicenne University Hospital, AP-HP, Bobigny, France; 9 ECSTRA Team, Biostatistics and Clinical Epidemiology, UMR 1153 (Center of Epidemiology and Biostatistics, Sorbonne Paris Cité, CRESS), INSERM, Paris Diderot Sorbonne University, Paris, France

**Keywords:** Follow-up study, Intensive care unit, Post-intensive, Prognosis, Score, Solid cancers

## Abstract

**Objectives:**

To assess outcomes at hospital discharge and day-120 after intensive care unit (ICU) discharge among patients with solid cancer admitted to ICU and to identify characteristics associated with in-hospital and day-120 after ICU discharge mortalities.

**Design:**

International, multicenter, retrospective study.

**Setting:**

Five ICUs in France and Brazil, two located in cancer centers, two in university affiliated and one in general hospitals.

**Patients:**

Consecutive patients aged > 18 years, with underlying solid cancers (known before admission to the ICU or diagnosed during the stay in the ICU), admitted to the participating ICUs and discharged alive from the ICU from January 2006 to December 2011 were included in this study. Patients admitted after scheduled surgery or to secure procedure were excluded. Variables of interest were in-hospital and day-120 post-ICU mortality among patients discharged alive from the ICU.

**Interventions:**

None.

**Measurements and results:**

A total of 1053 patients aged 63 years (54–71) (median [IQR]) were included. Most of the patients were of the male gender (66.8%). The in-ICU, in-hospital, and four-month post-ICU discharge mortalities were, respectively, 41.3, 60.7, and 65.8%. Among patients discharged alive from the ICU, in multivariate analysis, factors associated with four months post-ICU discharge mortality were type of cancer (OR from 0.25 to 0.52 when compared to lung cancers), systemic extension of the disease (OR 2.54; 95% CI 1.87–3.45), need for invasive mechanical ventilation (OR 2.54; 95% CI 1.80–3.59), for vasopressors (OR 2.35; 95% CI 1.66–3.29), or renal replacement therapy (OR 1.54; 95% CI 0.99–2.38). A predictive score, “Oncoscore,” was built performing fairly in predicting 4 months post-ICU discharge outcome (AUC 0.74; 95% CI 0.71–0.77).

**Conclusion:**

Despite the high day-120 mortality following the ICU discharge, our study reports a meaningful medium-term survival rate after the ICU discharge of solid cancer patients. Of utmost importance, the “Oncoscore” must be validated in prospective studies and cannot be used, in its form without external validation, for individual decision making. Prospective studies to answer questions not provided by this study are needed, including only patients with solid cancers admitted in the ICU for medical reasons or after emergency surgery.

**Electronic supplementary material:**

The online version of this article (10.1186/s13613-018-0386-6) contains supplementary material, which is available to authorized users.

## Background

Survival of patients with cancer has substantially increased in recent decades in developed countries, which, consequently, has led to increasing number of hospitalizations in the intensive care unit (ICU) [1, 2]. A retrospective study showed that among 118,541 patients, 5.2% (95% confidence interval [95% CI] 5.0–5.3%) were admitted to an ICU within 2 years following cancer diagnosis [3]. Despite this, data regarding in-hospital mortality and mainly post-ICU outcome of such patients remain scarce. In a literature review of adult patients with solid cancers admitted to the ICU from January 2000 to April 2014, Puxty et al. [4] identified 48 studies, among which in-ICU mortality was reported in 32 studies, in-hospital mortality reported in 25 studies, and mortality at 1, 3, and 6 months, respectively, reported in only 4, 5, and 7 studies. One of the most obvious reasons is that studies on cancer patients hospitalized in the ICU are still ongoing, and even the most recent ones continue to mix patients with hematological malignancies and those with solid cancers under the generic term “cancer patients” [5, 6]. However, there is evidence for several years that the prognosis of solid cancer patients is totally different depending on the type of cancer, whether hematological or solid [7]. One of the most recent and best documented studies is probably that of Ha et al. [8]. Its main interest lies in the fact that the population studied (unplanned admissions) was similar to ours.

The primary objective of our study was to assess hospital mortality of critically ill patients with solid cancers. The secondary objectives were to assess post-ICU day-120 mortality, to assess risk factors for poor outcomes in this population, and to develop a prediction score, named “Oncoscore,” regarding post-ICU day-120 mortality.

## Patients and methods

### Patients and centers

This retrospective cohort study was performed in five ICUs (two cancer institute ICUs [Instituto Nacional de Câncer, Rio de Janeiro and Paoli-Calmettes Institute, Marseille], two university-affiliated hospital ICUs [Saint-Louis Hospital, Paris and Avicenne Hospital, Bobigny], and one general hospital ICU [Versailles Hospital, Le Chesnay]). Consecutive patients aged > 18 years, with underlying solid cancers (known before admission to the ICU or diagnosed during the stay in the ICU), admitted to the participating centers from January 2006 to December 2011 and discharged alive from the ICU were included in this study. Cancer patients admitted following a scheduled surgery [9], those admitted to secure a procedure, or patients with prostate cancer (to avoid the debate on the overdiagnosis of this cancer) [10] were excluded from this study. Thus, only consecutive patients with solid cancers admitted for a medical reason or after emergency surgery were included. To analyze the vital status 4 months following ICU discharge, day 1 was considered as the day of ICU discharge.

In case of multiple admissions in the ICU, only the first one was considered.

According to the French law (L.1121-1 paragraph 1 and R1121-2, Public Health Code), informed consent was unnecessary for anonymous data extraction and analysis from patients’ medical files. This retrospective study was approved by the Ethics Commission of the French Intensive Care Society (CE SRLF16-49).

### Data collection and definitions

Variables collected within the first 24 h of ICU admission included age, gender, type of cancer, cancer extension (localized or with distant metastases [“systemic extension”]), cancer status (controlled or remission, uncontrolled or new diagnosis, uncontrolled or disease progression), primary reason for ICU admission, hospital days before ICU admission, simplified acute physiology score (SAPS II) [11], sequential organ failure assessment (SOFA) score [12], need for IMV, use of any vasopressors, and need for renal replacement therapy (RRT). During the ICU stay, details regarding the need for IMV, vasopressors, RRT, occurrence of neutropenia (neutrophils < 0.5 G/L), and do not resuscitate (DNR) codes were collected daily. The ICU length of stay (LOS), hospital LOS, vital status at the ICU and hospital discharges, and vital status 4 months following ICU discharges were collected for each patient.

### Statistical analysis

Results are reported as medians and quartiles (interquartile range, IQR) or numbers (%). Categorical variables were compared using the Chi-square test or Fisher’s exact test, as appropriate, and continuous variables were compared using the nonparametric Wilcoxon test or the Mann–Whitney test.

Logistic regression analyses were performed to identify the variables statistically significantly associated with hospital mortality and day-120 post-ICU discharge mortality, as measured by the estimated odds ratio (OR) with 95% CIs. Variables yielding *P* values < 0.20 in the univariate analyses or considered clinically relevant were entered in a forward stepwise logistic regression model. Non-log-linear continuous variables were dichotomized. The covariates were entered in the model with critical entry and removal *P* values of 0.20 and 0.1, respectively. Multi-collinearity and interactions were tested. The Hosmer–Lemeshow test was used to check goodness-of-fit of the logistic regression.

A score, named “Oncoscore,” was then built based upon independent factors associated with post-ICU day-120 outcome. To assess its performance in distinguishing post-ICU day-120 outcome, we plotted the receiver operating characteristic (ROC) curves of the proportion of true positives against the proportion of false positives. Two cutoff values were then selected (sensitive and specific cutoff) to define subgroups. Survival curves were then constructed according to the Kaplan–Meier method. Comparison across prediction class was performed using log-rank test.

All tests were two sided, and *P* values < 0.05 were considered statistically significant. Statistical tests were performed using SPSS 13 software package (IBM, Armonk, NY, USA).

## Results

A total of 1248 patients with solid cancers were screened during the study period. Of those, 195 had missing data and were excluded. Thus, 1053 were included in the study. The characteristics of the included patients are reported in Table 1.

**Table 1:** Patients characteristics per hospital outcome

Variable	Hospital decedents (*n* = 639)	Hospital survivors (*n* = 414)	*P* value
Male gender	404 (63.2%)	287 (69.3%)	0.05
Age (years)	63 [55–71]	63 [53–71]	0.29
Type of malignancy			
Lung	157 (24.6%)	45 (10.9%)	< 0.001
Colorectal	90 (14.1%)	88 (21.3%)	
Breast	68 (10.6%)	61 (14.7%)	
Head and neck	79 (12.4%)	46 (11.1%)	
Others	245 (38.4%)	174 (42.0%)	
Diagnosis to ICU (months)	3 [1–14]	6 [1–36]	
Cancer status			0.002
Complete remission	99 (15.5%)	90 (21.8%)	–
Partial remission	92 (14.4%)	51 (12.4%)	0.004
Diagnosis during ICU stay	52 (13.0%)	19 (7.5%)	0.04
Distant metastases	384 (60.1%)	177 (42.8%)	< 0.001
Severity at ICU admission			
SAPS II	54 [42–72]	39 [30–50]	< 0.001
SOFA	8 [5–12]	4 [2–7]	< 0.001
Treatments during ICU stay			
Invasive mechanical ventilation	518 (81.3%)	203 (49.0%)	< 0.001
Vasopressors and/or inotropic drug	403 (63.1%)	126 (30.5%)	< 0.001
Renal replacement therapy	127 (19.9%)	41 (10.0%)	< 0.001
Cancer chemotherapy	27 (4.4%)	13 (3.3%)	0.48
Neutropenia during ICU stay	52 (8.2%)	18 (4.4%)	0.02

Six hundred and ninety one (65.6%) were males with a median age of 63 years (range 54–71). Initial severity scores according to the SOFA and SAPS II scores were 6 (3–10) and 48 (36–62), respectively. The primary underlying malignancies were lung cancers in 202 (19.2%), colorectal cancer in 178 (16.9%), breast cancer in 129 (12.5%), and head and neck cancer in 125 (11.9%). Cancer was considered to be in partial or complete remission in 332 (31.5%) and was diagnosed during the ICU stay in 71 (6.7%). Overall, 561 (53.3%) had systemic extension (presence of distant metastasis) of their disease. Acute condition leading to ICU admission was considered as related to cancer status in 350 (33.3%).

The primary reasons for ICU admission were sepsis or septic shock in 405 (38.5%), acute respiratory failure in 275 (26.1%), and coma in 80 (7.6%). Seven hundred and twenty one (68.5%) required mechanical ventilation, 529 (50.2%) required vasopressors, and 168 (16.0%) required RRT. Cancer chemotherapy was required during ICU stay in 40 (3.8%), and 70 (6.6%) experienced neutropenia during their ICU stay.

### Hospital outcome

Survival after the ICU discharge was, respectively, for in-hospital 413/618 (66.8%) and for 120-days 360/618 (58.2%) (Additional file 1: Fig. S1). Factors independently associated with in-hospital mortality are reported in Table 2. After adjustment, lung cancers (when compared to other underlying malignancies), systemic extension of the disease (OR 2.64; 95% CI 1.95–3.57), and organ support (namely need for renal replacement therapy [RRT], vasopressors, or IMV (OR 1.65 [95% CI 1.08–2.53], OR 2.55 [95% CI 1.84–3.55] and OR 2.81 [95% CI 2.00–3.95]), respectively) were associated with poor outcome.

**Table 2:** Independent predictors of in-hospital mortality after discharged alive from the ICUs (conditional forward logistic regression)

Variable	Odds ratio	95% CI	*P* value
Systemic extension of the disease	2.54	1.87–3.45	< 0.001
Underlying tumor			
Lung cancers	Ref.	–	–
Colonic cancer	0.29	0.17–0.48	< 0.001
Breast cancer	0.25	0.14–0.43	< 0.001
Head and neck	0.52	0.29–0.93	0.03
Other	0.52	0.33–0.82	0.004
Renal replacement therapy during ICU	1.54	0.99–2.38	0.05
Vasopressors	2.35	1.66–3.29	< 0.0001
Mechanical ventilation	2.54	1.80–3.59	< 0.0001

Hosmer–Lemeshow goodness-of-fit: *k*^2^ = 1.90; *P* = 0.98. C-stat: 0.70

*CI* confidence interval, *Ref.* reference

During the ICU stay, decisions to forgo life-sustaining therapies were taken for 362 patients (34.4%). They were not selected in the first multivariable model. However, when these decisions were forced in the final model, these variables did not change this later.

### Day-120 after the ICUs discharge outcome

Overall, of the 618 ICU survivors, 258 (41.7%) died before day 120 (Additional file 1: Fig. S1 and Additional file 2: Fig. S2). Factors independently associated with poor day-120 outcome are reported in Table 2 and were found to be consistent with factors associated with hospital outcome. Of note, lung cancers remained associated with poor 4-month mortality in ICU survivors (Additional file 2: Fig. S2). By doing the analysis by center, no “center-effect” appeared.

### “Oncoscore”

“Oncoscore” ranges from 0 to 11 (Table 3). In the studied population, the median “Oncoscore” was 6 (range 3–7). The overall model area under the ROC curve in predicting day-120 outcome was fair (0.74; 95% CI 0.71–0.77) (Additional file 3: Fig. S3). Two cutoff values were assessed, including a sensitive cutoff (score of 4, sensitivity 0.84) and a specific cutoff (score of 8; specificity 0.92). According to these cutoff values, day-120 mortality rates were 40% (*n* = 111), 70% (*n* = 363), and 87% (*n* = 216) in patients with “Oncoscore” < 4, between 4 and 7, and ≥ 8, respectively (Fig. 2).

**Table 3:** Calculation of the Oncoscore

Characteristics	Points
Type of cancer	
Lung	2
Breast or colorectal	0
Others	1
Presence of distant metastasis	
No	0
Yes	2
Type of organ support received in the intensive care unit	
Invasive mechanical ventilation	3
Vasoactive and/or inotropic drug(s) (whatever the type)	2
Renal replacement therapy (whatever the type)	2

## Discussion

Studies that specifically focus on patients with solid cancers in the ICU are scarce, especially those which considered after-ICU survival [4]. Most of them are unicentric or based on databases and primarily focused on patients with lung cancers. In addition, they often only concern one type of cancer and mix patients admitted for scheduled surgery surveillance and those admitted after urgent surgery or for medical reasons that have absolutely no same prognosis in the short, medium, or long term. The largest to date retrospectively analyzed the unplanned admissions of 12,290 patients with solid cancers in 80 ICUs in the Netherlands over a 4-year period [5]. About 59.3% of all admissions were surgical (albeit unplanned), and these had a mortality (9.0 vs. 8.9% in the ICU and 17.4 vs. 14.6% in the hospital) like that of patients with no cancer diagnosis. Medical patients with cancer, however, had higher mortalities (30.4 vs. 16.2% in the ICU and 44.6 vs. 23.7% in the hospital). There was also a difference in outcomes for medical patients per cancer diagnosis; respiratory tract cancers were associated with an OR of 2.15 for ICU death, and upper gastrointestinal cancers were associated with an OR of 1.42. Among 35,308 Medicare beneficiaries who survived the ICU stay, the 6-month mortality was influenced by the existence of solid tumors without metastasis (adjusted hazard ratio [AHR] 1.15; 95% CI 1.08–1.23) [13]. The most important comorbidity, which determined the 6-month mortality after ICU discharge, was solid metastatic cancer (AHR 3.31; 95% CI 3.06–3.51). To the best of our knowledge, only three studies have questioned about the quality of life (QOL) of oncology patients after ICU discharge [14–16]. Only eight studies (six concerning patients with lung cancers) have reported the rates of patients who could receive anticancer treatment after a stay in the ICU [17–24].

The influence of ICU stay on the nature of subsequent antineoplastic therapy was evaluated in three studies, with change rates between 31 and 34% in relation to the project defined before the stay in the ICU [20, 23, 24].

**Fig. 1:**
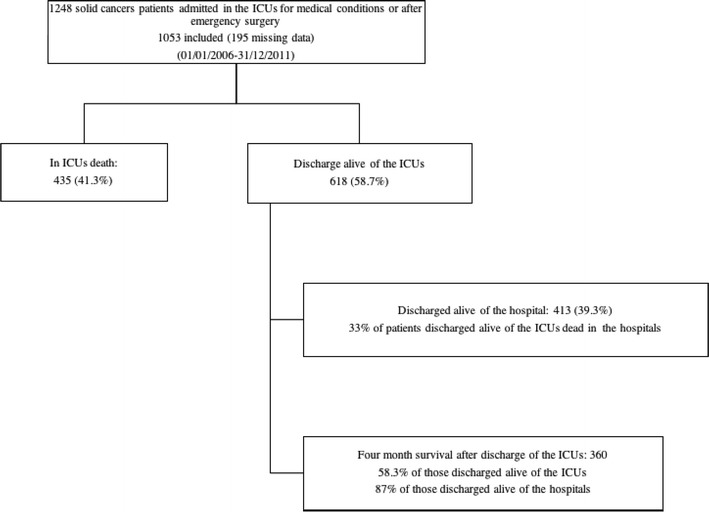
Four-month follow-up, after ICU discharge, of the 1053 patients included in the study

**Fig. 2:**
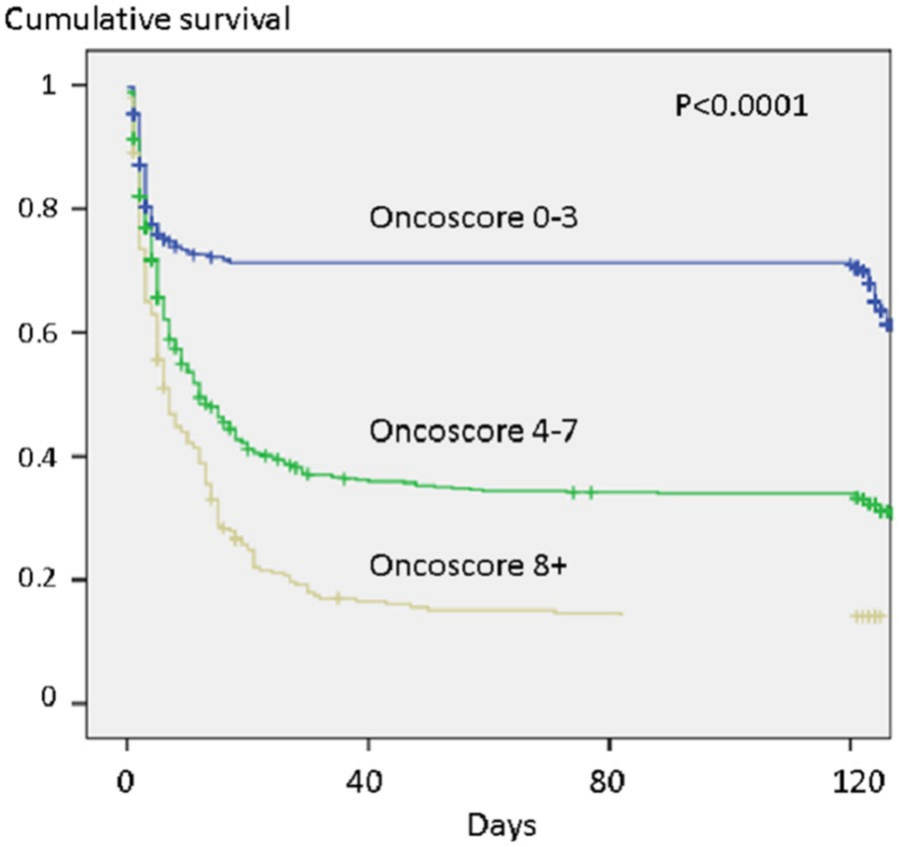
Cumulative 4 months after ICU discharge survival per the value of the Oncoscore (the overall model area under ROC curve in predicting day-120 post-ICU outcome was fair [0.74; 95% CI 0.71–0.77]. A score of 4 was found to be sensitive of poor outcome [sensitivity 0.84], while a score of 8 was specific [specificity 0.92]).

Our findings report some data on a poorly studied subject. Contrary to what is observed in patients with hematological malignancies, our findings suggest that cancer characteristics (type and remote location) influence the in-hospital and four-month survival [25]. Other elements seem specific to patients with solid cancers. The first one is the primary reason for admission, which, in our study, is sepsis or septic shock, while acute respiratory failure is the first one in large sets of hematology patients admitted to the ICU [26]. Second, our findings suggest that cancer characteristics influence the short- and medium-term survival in contrast to hematology patients [4, 27, 28]. This contrasts with the fact that other characteristics of cancer such as status previous specific treatment and age of diagnosis do not seem to influence the 4-month mortality. For this reason, these two parameters should substantially influence ICU triage decisions in patients with solid cancers as suggested by the “Oncoscore.” Third, few patients leaving the ICU alive died in the wards (3.3%). Moreover, the 4-month survival rate of 34.2% of all patients, among them, 360/618 (58.2%) who were discharged alive from the intensive care unit which was our variable of interest, is quite acceptable (Figs. 1, 2). These results, however, deserve to be modulated by not taking into account patients with lung cancers. Fourth, although some studies demonstrated a case–volume relationship in critically ill patients with hematological malignancies, it was not observed in our results [29]. Although it is, to our knowledge, the first to describe the medium-term outcome of “homogeneous” critically ill oncology patients (excluding those hospitalized in the ICU for scheduled surgery surveillance, i.e., planned admissions), our study has many limitations. First, it is a retrospective, multicenter, and international study. Though standard definitions were used, biases related to differences in data collection between the centers cannot be ruled out. Second, the choice of 4 months follow-up after the ICU discharge is debatable. Third, like most of the studies performed on this subject, the retrospective observational design does not describe data regarding patients who were not referred to the ICU, possibly introducing a significant selection bias. Fourth, we reported only crude mortality. A variable of interest among patients emerging alive from the ICU and hospital should probably be the possibility of reinstating optimal oncological treatment course. Fifth, we were not able to collect the Eastern Cooperative Oncology Group performance status (ECOG PS) score, or other one, on admission or on discharge of the ICU [30]; however, numerous studies on critically ill oncology patients suggest that this is one of the key determinants of short-term and medium-term survivals [4, 31]. Sixth, our data are simply quantitative and not qualitative. Seventh, it should be underlined that Oncoscore cannot be used to support individual decision making with respect to defining adequate goals of therapy in any form. We just postulated that the clinical and therapeutic data observed in the ICU could influence survival at 4 months after the ICU discharge. It should probably be adjusted regularly to reflect the advances in oncology. Finally, we believed that the prognosis of oncologic patients admitted in the ICU remains to be described and that improvement pathways are needed, as in patients with malignant hematological diseases [32]. Prospective studies to answer questions not provided by our one including only patients with solid cancers admitted in the ICU for medical reasons or after emergency surgery are urgently needed [8].

## Conclusion

The challenge for intensivists is to identify which patients with solid cancers would benefit from ICU cares. Our data, although reporting a high mortality in the ICU and in the hospital, show that the medium-term survival of these patients is quite acceptable, especially for patients with cancers other than lung cancers. The Oncoscore must be validated in a prospective cohort.

## Additional files

**Additional file 1: Fig. S1.** Four-months survival, after ICU discharge, of the 1053 patients included in the study.

**Additional file 2: Fig. S2.** Cumulative 4 months after ICU discharge survival in patients with solid cancers surviving to ICU stay and per the underlying disease. Patients with lung cancers had a poorer prognosis (log-rank test; *P* = 0.002).

**Additional file 3: Fig. S3.** Overall model area under ROC curve of Oncoscore in predicting day-120 outcome after ICU discharge (AUC ROC Curve = 0.74 [95% CI 0.71–0.77]).

## Abbreviations

AUC: area under the curve
CE SRLF: Ethics Commission of the French Intensive Care Society
ECOG PS: Eastern Cooperative Oncology Group performance status
HR: hazard ratio
ICU: intensive care unit
OR: odds ratio
QOL: quality of life
ROC curve: receiver operating characteristic curve
95% CI: 95% interval of confidence
