# Supplementary material for: In-hospital and day-120 survival of critically ill solid cancer patients after discharge of the intensive care units: results of a retrospective multicenter study—A Groupe de recherche respiratoire en réanimation en Onco–Hématologie (Grrr-OH) study
Source: Ann Intensive Care. 2018 Mar 27;8:40. doi: 10.1186/s13613-018-0386-6 (PMC6890921; doi:10.1186/s13613-018-0386-6)
Supplement: Supplementary file 1 — Additional file 1: Fig. S1. Four-months survival, after ICU discharge, of the 1053 patients included in the study. [file 13613_2018_386_MOESM1_ESM.docx]

**Figure S1: Four-months overall survival, after ICU-discharge, of the 1053 patients included in the study.**


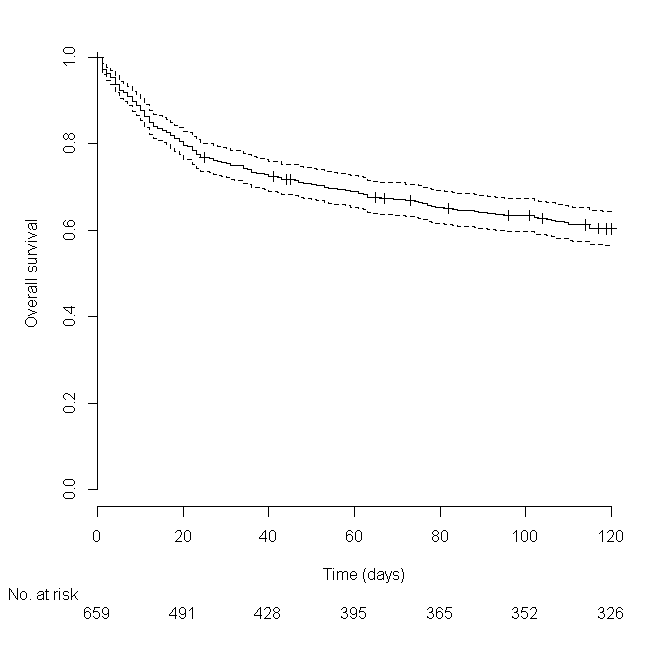


Day 0 corresponds to discharge of the Intensive Care Units.
