# Supplementary material for: In-hospital and day-120 survival of critically ill solid cancer patients after discharge of the intensive care units: results of a retrospective multicenter study—A Groupe de recherche respiratoire en réanimation en Onco–Hématologie (Grrr-OH) study
Source: Ann Intensive Care. 2018 Mar 27;8:40. doi: 10.1186/s13613-018-0386-6 (PMC6890921; doi:10.1186/s13613-018-0386-6)
Supplement: Supplementary file 2 — Additional file 2: Fig. S2. Cumulative 4 months after ICU discharge survival in patients with solid cancers surviving to ICU stay and per the underlying disease. Patients with lung cancers had a poorer prognosis (log-rank test; P = 0.002). [file 13613_2018_386_MOESM2_ESM.docx]

**Figure S2: Cumulative four-months after ICU discharge survival in patients with solid cancers surviving to ICU stay and per the underlying disease. Patients with lung cancers had a poorer prognosis (log-rank test; P=0.002).**

**
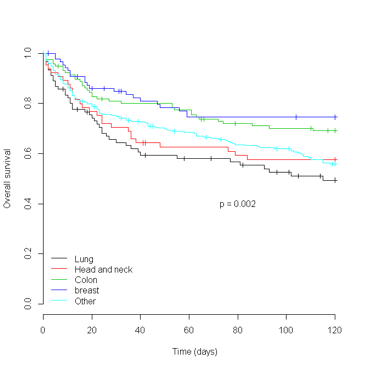
**

Day 0 corresponds to discharge of the Intensive Care Units.
