# Supplementary material for: In-hospital and day-120 survival of critically ill solid cancer patients after discharge of the intensive care units: results of a retrospective multicenter study—A Groupe de recherche respiratoire en réanimation en Onco–Hématologie (Grrr-OH) study
Source: Ann Intensive Care. 2018 Mar 27;8:40. doi: 10.1186/s13613-018-0386-6 (PMC6890921; doi:10.1186/s13613-018-0386-6)
Supplement: Supplementary file 3 — Additional file 3: Fig. S3. Overall model area under ROC curve of Oncoscore in predicting day-120 outcome after ICU discharge (AUC ROC Curve = 0.74 [95% CI 0.71–0.77]). [file 13613_2018_386_MOESM3_ESM.docx]

**Figure S3: Overall model area under ROC curve of Oncoscore in predicting day-120 outcome (AUC ROC Curve = 0.74 [95%CI: 0.71-0.77]).**
